# A103 DESIGNING QUALITATIVE RESEARCH FOR UNDERSTANDING THE EXPERIENCES OF TRANGENDER AND GENDER DIVERSE PERSONS WITH GASTROINTESTINAL DISEASE OR SEEKING GASTROINTESTINAL CARE

**DOI:** 10.1093/jcag/gwac036.103

**Published:** 2023-03-07

**Authors:** N Kariyawasam, K Newman, C G Streed, L Rizkalla, C Vélez, L Targownik

**Affiliations:** 1 University of Toronto, Toronto, Canada; 2 University of Michigan, Ann Arbor, Ann Arbor; 3 School of Medicine, Boston University; 4 Center for Transgender Medicine & Surgery, Boston Medical Center, Boston, United States; 5 None, Calgary, Canada; 6 Division of Gastroenterology, Massachusetts General Hospital, Boston, United States; 7 Mount Sinai Hospital, Toronto, Canada

## Abstract

**Background:**

Transgender and gender diverse (TGD) people make up approximately 1% of the population, with 2.1% of Generation Z adults (born 1997-2003) identifying as TGD. As a marginalized population, TGD people have been shown to have poorer access to health care services and report worse health-related outcomes. While the health care disparities faced by TGD people need to be addressed by Medicine broadly, TGD people may face unique barriers in gastrointestinal (GI) care. To date, there has been no systematic assessment of the GI health care needs of TGD people. With the aging of Generation Z, GI care providers will increasingly be responsible for the care of TGD people, and it is important to understand their needs and experiences to ensure they receive appropriate and sensitive care.

**Purpose:**

Recognizing the absence of literature on the issues faced by TGD people with GI disease or seeking care for GI issues, we plan to use qualitative research methodology to aggregate and analyze narrative experiences of TGD people of diverse backgrounds, identities and experiences. Our research will focus on TGD people’s interactions with GI care providers, and on the experience of being a TGD person with GI disease or having sought evaluation of GI symptoms.

**Method:**

We will use a qualitative approach to gain a broad understanding of the experiences and perceptions of TGD people with: 1) established GI diseases, in particular IBD and DGBIs (disorders of gut-brain interaction), and 2) GI symptoms or concerns requiring GI investigations. We will use snowball sampling to reach out to TGD people in Canada and the US, with the aim of achieving diverse representation across gender identity, surgical status, age, race/ethnicity, and socioeconomic status. Participants will sit for semi-structured interviews to elicit narratives about their beliefs and experiences while living with GI disease and/or seeking care for GI issues. Interview transcripts will be subjected to objective and researcher-guided thematic analysis to identify commonalities and disparities in experiences and points-of-view.

**Result(s):**

Our proposed presentation will highlight our process in designing and initiating this project. We believe our methods and approach to this work is an important discussion and will help shape next steps in this field. We may also present some of the early results of our semi-structured interviews.

**Conclusion(s):**

This work will provide a foundation to guide further research into the process and outcomes of care for TGD people with GI disease and undergoing GI evaluations and will provide a framework to develop best practices for GI care providers pertaining to the care of TGD people.

**Please acknowledge all funding agencies by checking the applicable boxes below:**

None

**Disclosure of Interest:**

None Declared

